# Improved modified estimator for estimation of median using auxiliary information under simple random sampling

**DOI:** 10.1038/s41598-024-67189-1

**Published:** 2024-07-17

**Authors:** Sohaib Ahmad, Saadia Masood, Abdullah Mohammed Alomair, Mohammed Ahmed Alomair

**Affiliations:** 1 Department of Statistics, Abdul Wali Khan University, Mardan, Pakistan; 2 Department of Mathematics and Statistics, PMAS University of Arid Agriculture, Rawalpindi, Pakistan; 3 Department of Quantitative Methods, School of Business, King Faisal University, 31982 Al-Ahsa, Saudi Arabia

**Keywords:** Median estimation, Auxiliary information, Simulation, Mean square error, Efficiency, Engineering, Mathematics and computing

## Abstract

This article aims to suggest an improved estimator for estimation of population median using auxiliary information under simple random sampling. The expression for the bias and mean square error are obtained up to first order approximation. We determine the MLE of the optimal values of the describing scalars. The proficiency of the suggested estimator is evaluated in comparison to the preliminary estimators using the MSE threshold. The suggested estimators are compared numerically to the ones that are currently studied in this study. The performance and novelty of the estimators was evaluated using real data sets and a simulation study. To check the efficiency of estimators empirical and theoretical study has been studied. Based on numerical result it is to be noted that our suggested estimator is more efficient as compared to existing estimators which is considered in this article in terms of least MSE and greater PRE.

## Introduction

In survey sampling, utilization of auxiliary information can improved estimator efficiency whichever at the design or at the estimation stage. Many efforts have been done for the purpose of increasing efficiency of an estimator. The efficient estimators of the population’s median have received comparatively less attention compared to that of its mean, proportion, variance, regression coefficient, etc., which have all been the subject of significant studies. Income, expenditure, taxation, consumption, and production are frequently of interest to researchers; at this point the distributions of these last two variables are extremely skewed. The median is a far better, rather than the mean in these circumstances. It is common for statisticians to be interested in handling variables with extremely skewed distributions, such earnings and consumption, in the sampling literature. In such circumstances, the median is believed to be a well central measure than the mean. The authors in Ref.^1^ recommended enhanced estimator for median exhausting auxiliary information under simple and stratified random sampling. Reference^2^ discussed difference type estimator for median estimation. The authors in Refs.^3–5^ discussed estimation of population median using two phase sampling. Reference^6^ recommended median estimator using auxiliary information. Reference^7^ suggested improved estimator for population median. References^8,9^ discussed a novel estimator utilizing auxiliary information. Reference^10^ recommend ratio and regression type estimator for population median. Reference^11^ suggested enhanced estimator for population median usig two phase sampling. The authors in Refs.^12,13^ discussed exponential and ratio type estimator for population median. Reference^14^ discussed difference type estimator for population median using auxiliary information. The authors in Refs.^15–24^ discussed some improved estimator for population parameter under different sampling design using auxiliary information.

## Materials and methods

Let a population consists $\text{W}$ = $\left({\text{W}}_{1}, {\text{W}}_{2}, \dots , {\text{W}}_{N}\right)$ of *N* differing units. Let $Y_{i}$ and $X_{i}$ be the $i{\text{th}}$ values of the study variable and the auxiliary variables. Let a sample of *n* is chosen from $\text{W}$. The sample and population medians of the study and the auxiliary variable are denoted by $M_{y}$ and $M_{x}$, and $M_{y}$ and $M_{x}$ with probability density functions of *fy* $\left({M}_{y}\right)$ and *fx* $\left({M}_{x}\right)$. The correlation coefficienct between the $M_{y}$ and $M_{x}$ are represented by ${\rho }_{yx}$ and is defined as ${\rho }_{\left({M}_{y}, {M}_{x}\right)}$ = 4 ${P}_{11}\left(y,x\right) -$ 1, where ${P}_{11}$ = $P\left(y\le {M}_{y}\cap x\le {M}_{x}\right)$.

To gain the expressions of bias and MSE we used the following error terms:

$${{e}_{y}}=\left(\frac{{{\widehat{M}}_{y}}-{{M}_{y}}}{{M}_{y}}\right), \text{ and }{{e}_{x}}=\left(\frac{{{\widehat{M}}_{x}}-{{M}_{x}}}{{M}_{x}}\right),$$

$${\text{E}}\left({e}_{y}^{2}\right)= \lambda {C}_{My}^{2},\text{ E}\left({e}_{x}^{2}\right)= \lambda {C}_{Mx}^{2}, {\text{E}}\left({e}_{y}\,{e}_{x} \right)=\lambda {C}_{Myx}^{2},$$

where

$${C}_{My}=\frac{1}{{M}_{y}\left({M}_{y}\right)}, {C}_{Mx}=\frac{1}{{M}_{x}{f}_{x}\left({M}_{x}\right)}, {C}_{Myx }={\rho }_{yx}{C}_{My}{C}_{Mx},\Lambda =\left(\frac{1}{4}\left\{\frac{1}{n}-\frac{1}{N}\right\}\right).$$

## Existing counterparts

This section consists of some well know existing estimators for comparison purpose.

1. Reference^6^ discuss usual unbiased median estimator $\left({\widehat{M}}_{y}\right)$, which is given by:
  1 $${\widehat{M}}_{y}={\widetilde{M}}_{y}.$$
  The variance of ${\widehat{M}}_{y}$, is given by:
  2 $$ Var\left( {\hat{M}_{y} } \right) = \Lambda M_{y}^{2} C_{My}^{2} = \Lambda \left\{ {f_{y} (M_{y} )} \right\}^{ - 2} . $$
2. Reference^9^ recommended ratio estimator, which is given by:
  3 $$ \hat{M}_{R} = \hat{M}_{y} \left( {\frac{{M_{x} }}{{M_{x} }}} \right). $$
  The expressions of $Bias\left( {\hat{M}_{R} } \right)$ and $MSE\left( {\hat{M}_{R} } \right)$ are given by:
  $$ Bias\left( {\hat{M}_{R} } \right) \cong \Lambda M_{y} \left( {C_{Mx}^{2} - C_{Myx} } \right), $$
  4 $$ MSE\left( {\hat{M}_{R} } \right) \cong \Lambda M_{y}^{2} \left( {C_{My}^{2} + C_{Mx}^{2} - 2C_{Myx} } \right). $$
3. The exponential ratio-type estimator is given by:
  5 $$ \hat{M}_{EX} = \hat{M}_{y} \exp \left( {\frac{{M_{x} - \hat{M}_{x} }}{{M_{x} + \hat{M}_{x} }}} \right). $$
  The expressions of $Bias\left( {\hat{M}_{EX} } \right)$ and $MSE\left( {\hat{M}_{EX} } \right)$ are given by
  $$ Bias\left( {\hat{M}_{EX} } \right) \cong \Lambda M_{y} \left( {\frac{3}{8}C_{Mx}^{2} - \frac{1}{2}C_{Myx} } \right), $$
  and
  6 $$ MSE\left( {\hat{M}_{EX} } \right) \cong \Lambda M_{y}^{2} \left( {C_{My}^{2} + \frac{1}{4}C_{Mx}^{2} - 2C_{Myx} } \right){.} $$
4. The difference estimator, is given by:
  7 $$ \hat{M}_{D} = \hat{M}_{y} + d\left( {M_{x} - \hat{M}_{x} } \right){.} $$
  The optimum value of *d* is given by:
  $$ d_{opt} = \frac{{M_{y} \rho_{yx} C_{My} }}{{M_{x} C_{Mx} }}. $$
  The minimum $MSE\left( {\hat{M}_{D} } \right)$ at the optimum value is given by:
  8 $$ MSE\left( {\hat{M}_{D} } \right)_{\min } \cong \Lambda M_{y}^{2} C_{My}^{2} \left( {1 - \rho_{xy}^{2} } \right){.} $$
5. Following^5,10,25^ respectively, proposed some difference-type estimators $\hat{M}_{Di} (i = 1,2,3)$, which is given by:
  9 $$ \hat{M}_{D1} = d_{1} \hat{M}_{y} + d_{2} \left( {M_{x} - \hat{M}_{x} } \right){,} $$
  10 $$ \hat{M}_{D2} = \left[ {d_{3} \hat{M}_{y} + d_{4} \left( {M_{x} - \hat{M}_{x} } \right)} \right]\left( {\frac{{M_{x} }}{{\hat{M}_{x} }}} \right), $$
  11 $$ \hat{M}_{D3} = \left[ {d_{5} \hat{M}_{y} + d_{6} \left( {M_{x} - \hat{M}_{x} } \right)} \right]\exp \left( {\frac{{M_{x} - \hat{M}_{x} }}{{M_{x} + \hat{M}_{x} }}} \right){.} $$
  At the optimum values of $d_{i} (i = 1,2,.....,6)$ the bias of $\hat{M}_{Di} (i = 1,2,3)$ are given by
  $$ Bias\left( {\hat{M}_{D1} } \right) \cong M_{y} \left( {d_{1} - 1} \right){,} $$
  $$ Bias\left( {\hat{M}_{D2} } \right) \cong M_{y} \left( {d_{3} - 1} \right) + d_{3} \Lambda M_{y} \left( {C_{Mx}^{2} - C_{Myx} } \right) + d_{4} \Lambda M_{x} C_{Mx}^{2} {,} $$
  $$ Bias\left( {\hat{M}_{D3} } \right) \cong M_{y} \left( {d_{5} - 1} \right) + d_{5} \Lambda M_{y} \left\{ {\frac{3}{8}C_{Mx}^{2} - \frac{1}{2}C_{Myx} } \right\} + \frac{1}{2}d_{6} \Lambda M_{x} C_{Mx}^{2} . $$
  At the optimum values of $d_{i} (i = 1,2,...,6)$
  $$ d_{1(opt)} = \frac{1}{{1 + \Lambda C_{My}^{2} \left( {1 - \rho_{xy}^{2} } \right)}},\;d_{5(opt)} = \frac{{1 - \left( {{{\Lambda C_{Mx}^{2} } \mathord{\left/ {\vphantom {{\Lambda C_{Mx}^{2} } 8}} \right. \kern-0pt} 8}} \right)}}{{1 + \Lambda C_{My}^{2} \left( {1 - \rho_{xy}^{2} } \right)}}, $$
  $$ d_{3(opt)} = \frac{{1 - \Lambda C_{Mx}^{2} }}{{1 - \Lambda C_{Mx}^{2} + \Lambda C_{My}^{2} \left( {1 - \rho_{xy}^{2} } \right)}},\;d_{2(opt)} = \frac{{M_{y} }}{{M_{x} }}\left[ {\frac{{\rho_{xy} C_{My} }}{{C_{Mx} \left\{ {1 + \Lambda C_{My}^{2} \left( {1 - \rho_{xy}^{2} } \right)} \right\}}}} \right], $$
  $$ d_{4(opt)} = \frac{{M_{y} }}{{M_{x} }}\left[ {1 + d_{3(opt)} \left( {\frac{{\rho_{xy} C_{My} }}{{C_{Mx} }} - 2} \right)} \right]\;{\text{and}}\;d_{6(opt)} = \frac{{M_{y} }}{{M_{x} }}\left[ {\frac{1}{2} + d_{5(opt)} \left( {\frac{{\rho_{xy} C_{My} }}{{C_{Mx} }} - 1} \right)} \right]. $$
  The minimum mean square error of $\hat{M}_{Di} (i = 1,2,3)$ are given by
  12 $$ MSE\left( {\hat{M}_{D1} } \right)_{\min } \cong \frac{{\Lambda M_{y}^{2} C_{My}^{2} \left( {1 - \rho_{xy}^{2} } \right)}}{{1 + \Lambda C_{My}^{2} \left( {1 - \rho_{xy}^{2} } \right)}}, $$
  13 $$ MSE\left( {\hat{M}_{D2} } \right)_{\min } \cong \frac{{\Lambda M_{y}^{2} \left( {1 - \Lambda C_{Mx}^{2} } \right)C_{My}^{2} \left( {1 - \rho_{xy}^{2} } \right)}}{{\left( {1 - \Lambda C_{Mx}^{2} } \right) + \Lambda C_{My}^{2} \left( {1 - \rho_{xy}^{2} } \right)}}{,} $$
  14 $$ MSE\left( {\hat{M}_{D3} } \right)_{\min } \cong \frac{{\Lambda M_{y}^{2} \left[ {C_{Mx}^{2} \left( {1 - \rho_{xy}^{2} } \right) - \frac{\Lambda }{4}C_{Mx}^{2} \left\{ {\frac{1}{16}C_{Mx}^{2} + C_{My}^{2} \left( {1 - \rho_{xy}^{2} } \right)} \right\}} \right]}}{{1 + \Lambda C_{My}^{2} \left( {1 - \rho_{xy}^{2} } \right)}}. $$
6. Reference^14^ suggested the generalized difference-type estimator for population median, which is given by:
  15 $$ \hat{M}^{G}_{pp} = \left[ {m_{1} \hat{M}_{y} + m_{2} \left( {M_{x} - \hat{M}_{x} } \right)} \right]\left[ {\left( {\frac{{aM_{x} + b}}{{a\hat{M}_{x} + b}}} \right)\exp \left\{ {\frac{{\alpha_{2} a\left( {M_{x} - \hat{M}_{x} } \right)}}{{a\left\{ {(\gamma - 1)M_{x} + \hat{M}_{x} } \right\} + 2b}}} \right\}} \right], $$
  where $m_{1}$ and $m_{2}$ are unidentified constants whose values are to be determined, $a$ and $b$ defined as unknown population parameters and $\alpha_{1}$, $\alpha_{2}$ and $\gamma$ are scalar quantities which can take different values like $\alpha_{1} = b = 0$ and $\alpha_{2} = \gamma = a = 1$.
  The $Bias\left( {\hat{M}^{G}_{pp} } \right)$ is given by
  $$ Bias\left( {\hat{M}_{pp}^{G} } \right) \cong (m_{1} - 1)M_{y} + m_{2} M_{y} \Lambda \left\{ {\frac{3}{2}C_{Mx}^{2} - C_{My} } \right\} + m_{2} M_{x} \Lambda C_{Mx}^{2} {.} $$
  At the optimum values of
  $$ m_{1(opt)} = \frac{{1 - 0.5\Lambda C_{Mx}^{2} }}{{1 + \Lambda C_{My}^{2} (1 - \rho_{xy}^{2} )}}, $$
  and
  $$ m_{2(opt)} = \frac{{M_{y} }}{{M_{x} }}\left[ {1 + m_{1(opt)} \left\{ {\frac{{\rho_{xy} C_{My} }}{{C_{Mx} }} - 2} \right\}} \right]. $$
  The $\min MSE\left( {\hat{M}^{G}_{pp} } \right)$ is given by
  16 $$ MSE\left( {\hat{M}^{G}_{pp} } \right)_{\min } \cong \frac{{M_{y}^{2} }}{{1 + \Lambda C_{My}^{2} (1 - \rho_{xy}^{2} )}}\left[ {\Lambda C_{My}^{2} (1 - \rho_{xy}^{2} ) - \frac{1}{4}\Lambda^{2} C_{Mx}^{4} - \Lambda^{2} C_{My}^{2} C_{Mx}^{2} (1 - \rho_{xy}^{2} )} \right]{.} $$
7. The author in Ref.^2^ proposed the following difference type estimator using two-auxiliary variables
  17 $$ \hat{M}_{P}^{I} = \left\{ {\hat{M}_{y} + m_{1} \left( {M_{x} - \hat{M}_{x} } \right)} \right\}\left\{ {m_{2} \exp \left( {\frac{{M_{z} - \hat{M}_{z} }}{{M_{z} + \hat{M}_{z} }}} \right)} \right.\left. { + \left( {1 - m_{2} } \right)\exp \left( {\frac{{\hat{M}_{z} - M_{z} }}{{M_{z} + \hat{M}_{z} }}} \right)} \right\}{.} $$
  The $Bias\left( {\hat{M}_{P}^{I} } \right)$ is given by
  $$ Bias\left( {\hat{M}_{P}^{I} } \right) = \Lambda \left[ {m_{1} M_{x} C_{Mxz} \left( {m_{2} - 0.5} \right) + M_{y} C_{Myz} \left( {0.5 - m_{2} } \right)} \right]. $$
  The $MSE\left( {\hat{M}_{P}^{I} } \right)$ is given by
  $$ MSE\left( {\hat{M}_{P}^{I} } \right) = \Lambda \left[ {M_{y}^{2} } \right.\left\{ {\left( {C_{My}^{2} + 0.25C_{Mz}^{2} } \right) + m_{2} C_{Mz} \left( {m_{2} } \right.C_{Mz} - C_{Mz} - 2\rho_{yz} \left. {C_{My} } \right) + \rho_{yz} C_{My} C_{Mz} } \right\} + m_{1} M_{x} C_{Mx} \left( {m_{1} } \right.\,M_{x} C_{Mx} - 2M_{y} \rho_{yx} C_{My} - \rho_{xz} C_{Mz} M_{y} + 2m_{2} \rho_{xz} C_{Mz} \left. {\left. {M_{y} } \right)} \right], $$
  $$ m_{1(opt)} = \frac{{C_{My} M_{y} \left( {\rho_{xz} \rho_{yz} - \rho_{yx} } \right)}}{{C_{Mx} M_{x} \left( {\rho_{xz}^{2} - 1} \right)}}, $$
  $$ m_{2(opt)} = \frac{{C_{Mz} \left( {\rho_{xz}^{2} - 1} \right) + 2C_{My} \left( {\rho_{xz} \rho_{yx} - \rho_{yz} } \right)}}{{2C_{Mz} \left( {\rho_{xz}^{2} - 1} \right)}}. $$
  At the optimum values of, the expression of $MSE(M_{p}^{I} )_{\min }$ is given by:
  18 $$ MSE(\hat{M}_{p}^{I} )_{\min } = \frac{{\Lambda M_{y}^{2} C_{My}^{2} }}{{\left( {1 - \rho_{xz}^{2} } \right)}}\left( {1 - \rho_{xz}^{2} - \rho_{yx}^{2} - \rho_{yz}^{2} + 2\rho_{yx} \rho_{xz} \rho_{yz} } \right). $$
8. Reference^26^ proposed the following ratio-cum-product exponential-type median estimator as
  19 $$ \hat{M}_{SM} = \hat{M}_{y} \left\{ {m_{12} \left( {\frac{{M_{x} }}{{\hat{M}_{x} }}} \right)} \right.\left. { + m_{13} \left( {\frac{{\hat{M}_{x} }}{{M_{x} }}} \right)} \right\}\left\{ {\alpha \exp \left( {\frac{{M_{x} - \hat{M}_{x} }}{{M_{x} + \hat{M}_{x} }}} \right)} \right.\left. { + \left( {1 - \alpha } \right)\exp \left( {\frac{{\hat{M}_{x} - M_{x} }}{{M_{x} + \hat{M}_{x} }}} \right)} \right\}{,} $$
  $$ Bias\left( {\hat{M}_{SM} } \right) = M_{y} \left[ {\left( {m_{12} + m_{13} - 1} \right) + m_{12} \Lambda \left\{ {\left( {\frac{3}{8} + \frac{3\alpha }{2}} \right)C_{Mx}^{2} - \left( {\alpha + \frac{1}{2}} \right)C_{Myx} } \right\}} \right.\left. { + m_{13} \Lambda \left\{ {\left( {\frac{3}{2} - \alpha } \right)C_{Myx} + \left( {\frac{3}{8} - \frac{\alpha }{2}} \right)C_{Mx}^{2} } \right\}} \right]. $$

The optimal values of $m_{12(opt)} = \left[ {\frac{{A_{2} A_{4} - A_{3} A_{5} }}{{A_{1} A_{2} - A_{3}^{2} }}} \right]$ and $m_{13(opt)} = \left[ {\frac{{A_{1} A_{5} - A_{3} A_{4} }}{{A_{1} A_{2} - A_{3}^{2} }}} \right]$, the expression of $MSE\left( {M_{SM} } \right)_{\min }$ is given by:

20 $$ MSE\left( {M_{SM} } \right)_{\min } = M_{y}^{2} \left[ {1 - \frac{{A_{1} A_{5}^{2} - 2A_{3} A_{4} A_{5} + A_{2} A_{4}^{2} }}{{A_{1} A_{2} - A_{3}^{2} }}} \right], $$

where $$A_{1} = 1 + \Lambda \left\{ {C_{My}^{2} + \left( {\alpha^{2} + 4\alpha + 1} \right)C_{Mx}^{2} - 2\left( {2\alpha + 1} \right)C_{Myx} } \right\},$$ $$A_{2} = 1 + \Lambda \left\{ {C_{My}^{2} + \left( {\alpha^{2} - 4\alpha + 3} \right)C_{Mx}^{2} + 2\left( {3 - 2\alpha } \right)C_{Myx} } \right\},$$ $$A_{3} = 1 + \Lambda \left\{ {C_{My}^{2} + 2\left( {1 - 2\alpha } \right)C_{Myx} + \alpha^{2} C_{Mx}^{2} } \right\},$$ $$A_{4} = 1 + \Lambda \left\{ {\left( {\frac{3}{8} + \frac{3\alpha }{2}} \right)C_{Mx}^{2} - \left( {\alpha + \frac{1}{2}} \right)C_{Myx} } \right\}$$ and $$A_{5} = 1 + \Lambda \left\{ {\left( {\frac{3}{8} - \frac{\alpha }{2}} \right)C_{Mx}^{2} + \left( {\frac{3}{2} - \alpha } \right)C_{Myx} } \right\}$$.

## Proposed estimator

Estimator performance can be enhanced by making use of auxiliary information during design and estimation stages. We used auxiliary information to determine the population median based on this notion. Taking motivation from Ref.^27^, we have suggested an improved estimator for population median using single auxiliary variable under simple random sampling. Our suggested estimator under simple random sampling offers several advantages over the existing estimators, the most notable of which are their enhanced adaptability, efficiency, and originality, which is given by:

21 $$ \hat{M}_{y}^{*} = \left[ {\alpha_{1} \hat{M}_{y} \left\{ {\frac{1}{2}\left( {\frac{{M_{x} }}{{\hat{M}_{x} }} + \frac{{\hat{M}_{x} }}{{M_{x} }}} \right)} \right\} + \alpha_{2} \left( {M_{x} - \hat{M}_{x} } \right)} \right]\exp \left( {\frac{{M_{x} - \hat{M}_{x} }}{{M_{x} + \hat{M}_{x} }}} \right). $$

By expressing the above equation in error terms, we have:

$$ \hat{M}_{y}^{*} = \left[ {\alpha_{1} M_{y} (1 + e_{y} )\left\{ {\frac{1}{2}\left( {(1 + e_{x} )^{ - 1} + 1 + e_{x} } \right)} \right\} + \alpha_{2} \left( {M_{x} - M_{x} (1 + e_{x} )} \right)} \right]\exp \left( {\frac{{M_{x} - M_{x} (1 + e_{x} )}}{{M_{x} + M_{x} (1 + e_{x} )}}} \right). $$

After simplifying we have:

$$ \hat{M}_{y}^{*} - M_{y} = \left[ {\alpha_{1} M_{y} \left\{ {1 + e_{y} + \frac{{e_{x}^{2} }}{2}} \right\} - \alpha_{2} M_{x} e_{x} } \right]\exp \left( {\frac{{ - e_{x} }}{{2 + e_{x} }}} \right) - M_{y} , $$

$$ \hat{M}_{y}^{*} - M_{y} = \left[ {\alpha_{1} M_{y} \left\{ {1 + e_{y} + \frac{{e_{x}^{2} }}{2}} \right\} - \alpha_{2} M_{x} e_{x} } \right]\exp \left\{ {\frac{{ - e_{x} }}{2}\left( {1 - \frac{{e_{x} }}{2} + \frac{{e_{x}^{2} }}{4}} \right)} \right\} - M_{y} , $$

$$ \hat{M}_{y}^{*} - M_{y} = \left[ {\alpha_{1} M_{y} \left\{ {1 + e_{y} + \frac{{e_{x}^{2} }}{2}} \right\} - \alpha_{2} M_{x} e_{x} } \right]\exp \left\{ {\left( {\frac{{e_{x}^{2} }}{4} - \frac{{e_{x} }}{2}} \right)} \right\} - M_{y} , $$

$$ \hat{M}_{y}^{*} - M_{y} = \left[ {\alpha_{1} M_{y} \left\{ {1 + e_{y} + \frac{{e_{x}^{2} }}{2}} \right\} - \alpha_{2} M_{x} e_{x} } \right]\left( {1 - \frac{{e_{x} }}{2} + \frac{{3e_{x}^{2} }}{8}} \right) - M_{y} , $$

$$ \hat{M}_{y}^{*} - M_{y} = \left[ {\alpha_{1} M_{y} \left\{ {1 + e_{y} + \frac{{e_{x}^{2} }}{2}} \right\}\left( {1 - \frac{{e_{x} }}{2} + \frac{{3e_{x}^{2} }}{8}} \right) - \alpha_{2} M_{x} \left( {e_{x} - \frac{{e_{x}^{2} }}{2}} \right)} \right] - M_{y} , $$

$$ \hat{M}_{y}^{*} - M_{y} = \left[ {\alpha_{1} M_{y} \left\{ {1\left( {1 - \frac{{e_{x} }}{2} + \frac{{3e_{x}^{2} }}{8}} \right) + e_{y} \left( {1 - \frac{{e_{x} }}{2} + \frac{{3e_{x}^{2} }}{8}} \right) + \frac{{e_{x}^{2} }}{2}\left( {1 - \frac{{e_{x} }}{2} + \frac{{3e_{x}^{2} }}{8}} \right)} \right\} - \alpha_{2} M_{x} \left( {e_{x} - \frac{{e_{x}^{2} }}{2}} \right)} \right] - M_{y} , $$

$$ \hat{M}_{y}^{*} - M_{y} = \left[ {\alpha_{1} M_{y} \left\{ {1 + e_{y} - \frac{{e_{x} }}{2} + \frac{{7e_{x}^{2} }}{8} - \frac{{e_{x} e_{y} }}{2}} \right\} - \alpha_{2} M_{x} \left( {e_{x} - \frac{{e_{x}^{2} }}{2}} \right)} \right] - M_{y} , $$

22 $${M}_{y}^{*}-{M}_{y}=\left({\alpha }_{1}\times {M}_{y}\times \left(1+{e}_{y}-\frac{{e}_{x}}{2}+\frac{7\times {e}_{x}\times {e}_{x}}{8}-\frac{{e}_{x}\times {e}_{y}}{2}\right)+{\alpha }_{2}\times {M}_{x}\times \left(\frac{{e}_{x}\times {e}_{x}}{2}-{e}_{x}\right)-{M}_{y}\right).$$

Taking expectation on both sides of Eq. (22), we have:

$${Bias(M}_{\text{y}}^{*})=E\left[{\alpha }_{1}\times {M}_{y}\times \left(1+{e}_{y}-\frac{{e}_{x}}{2}+\frac{7\times {e}_{x}\times {e}_{x}}{8}-\frac{{e}_{x}\times {e}_{y}}{2}\right)+{\alpha }_{2}\times {M}_{x}\times \left(\frac{{e}_{x}\times {e}_{x}}{2}-{e}_{x}\right)-{M}_{y}\right],$$

$$\text{Bias}\left({M}_{\text{y}}^{*}\right)=\left[{\alpha }_{1}\times {M}_{y}\times \left(1+\frac{7\times \varphi \times {C}_{x}^{2}}{8}-\frac{\varphi \times {R}_{\text{yx}}{C}_{y}{C}_{x}}{2}\right)+{\alpha }_{2}\times {M}_{x}\times \left(\frac{\varphi \times {C}_{x}^{2}}{2}\right)-{M}_{y}\right].$$

Squaring Eq. (22), and ignoring higher order terms and taking expectation, we got:

$$\text{MSE}\left({M}_{\text{y}}^{*}\right)=E{\left[{\alpha }_{1}\times {M}_{y}\times \left(1+{e}_{y}-\frac{{e}_{x}}{2}+\frac{7\times {e}_{x}\times {e}_{x}}{8}-\frac{{e}_{x}\times {e}_{y}}{2}\right)+{\alpha }_{2}\times {M}_{x}\times \left(\frac{{e}_{x}\times {e}_{x}}{2}-{e}_{x}\right)-{M}_{y}\right]}^{2},$$

$$\text{MSE}\left({M}_{\text{y}}^{*}\right)=\left[{M}_{y}^{2}-2{M}_{y}^{2}{\alpha }_{1}-\frac{7}{4}{e}_{x}^{2}{M}_{y}^{2}{\alpha }_{1}+{e}_{x}{e}_{y}{M}_{y}^{2}{\alpha }_{1}+{M}_{y}^{2}{\alpha }_{1}^{2}+2{e}_{x}^{2}{M}_{y}^{2}{\alpha }_{1}^{2}-2{e}_{x}{e}_{y}{M}_{y}^{2}{\alpha }_{1}^{2}+{e}_{y}^{2}{M}_{y}^{2}{\alpha }_{1}^{2}-{e}_{x}^{2}{M}_{x}{M}_{y}{\alpha }_{2}+2{e}_{x}^{2}{M}_{x}{M}_{y}{\alpha }_{1}{\alpha }_{2}-2{e}_{x}{e}_{y}{M}_{x}{M}_{y}{\alpha }_{1}{\alpha }_{2}+{e}_{x}^{2}{M}_{x}^{2}{\alpha }_{2}^{2}\right],$$

23 $$\text{MSE}\left({M}_{\text{y}}^{*}\right)={M}_{y}^{2}-2{M}_{y}^{2}{\alpha }_{1}-\frac{7}{4}\varphi \times {C}_{x}^{2}\times {M}_{y}^{2}{\alpha }_{1}+\varphi \times {R}_{\text{yx}}{C}_{y}{C}_{x}{M}_{y}^{2}{\alpha }_{1}+{M}_{y}^{2}{\alpha }_{1}^{2}+2\varphi \times {C}_{x}^{2}{M}_{y}^{2}{\alpha }_{1}^{2}-2\varphi \times {R}_{\text{yx}}{C}_{y}{C}_{x}{M}_{y}^{2}{\alpha }_{1}^{2}+\varphi \times {C}_{y}^{2}{M}_{y}^{2}{\alpha }_{1}^{2}-\varphi \times {C}_{x}^{2}{M}_{x}{M}_{y}{\alpha }_{2}+2\varphi \times {C}_{x}^{2}{M}_{x}{M}_{y}{\alpha }_{1}{\alpha }_{2}-2\varphi \times {R}_{\text{yx}}{C}_{y}{C}_{x}{M}_{x}{M}_{y}{\alpha }_{1}{\alpha }_{2}+\varphi \times {C}_{x}^{2}\times {M}_{x}^{2}{\alpha }_{2}^{2},$$

$${\alpha }_{1opt}=\frac{8+3\varphi {C}_{x}^{2}}{8(1+\varphi {C}_{x}^{2}+{C}_{y}^{2}(\varphi -\varphi {R}_{\text{yx}}^{2}))},$$

$${\alpha }_{2opt}=\frac{{M}_{y}(\varphi {C}_{x}^{3}+8{C}_{y}{R}_{\text{yx}}+3\varphi {C}_{x}^{2}{C}_{y}{R}_{\text{yx}}-4{C}_{x}(1+\varphi {C}_{y}^{2}(-1+{R}_{\text{yx}}^{2})))}{8{C}_{x}{M}_{x}(1+\varphi {C}_{x}^{2}+{C}_{y}^{2}(\varphi -\varphi {R}_{\text{yx}}^{2}))}.$$

Putting the optimum values of ${\alpha }_{1opt}$ and ${\alpha }_{2opt}$ in Eq. (23), we got the minimum mean square, we have:

24 $$\text{MSE}{({M}_{\text{y}}^{*})}_{\text{min}}={M}_{y}^{2}-\frac{{M}_{y}^{2}\left[64+\varphi {C}_{x}^{2}(64+\varphi \left\{25{C}_{x}^{2}-16{C}_{y}^{2}\left(-1+{R}_{\text{yx}}^{2}\right)\right\}\right]}{64\left[1+\varphi {C}_{x}^{2}+\varphi {C}_{y}^{2}\left\{1-{R}_{\text{yx}}^{2}\right\}\right]}.$$

## Numerical study

In this section, we take three actual data sets to observe the efficiency of an estimators. Here, we consider three actual data sets. The PRE of ${\widehat{M}}_{i}$ with respect to ${\widehat{M}}_{y}$ is carried using below expression.

$${\text{PRE}}=\frac{\text{Var}\left({\widehat{M}}_{y}\right) }{MSE\left({\widehat{M}}_{i}\right)}\times 100.$$

Population 1: [Source^28^].

*Y* = Number of faculty, *X* = Number of students in 4 diverse schools under 36 districts in Punjab.

*N* = 144, *n* = 10, $$\Lambda$$ = 0.02327, $${M}_{y}$$ = 2023, $${M}_{x}$$ = 64,659, *fy* $$\left({M}_{y}\right)$$ = 0.00024, *fx* $$\left({M}_{x}\right)$$ = 0.00001, $${\rho }_{yx}$$ = 0.86110, $$C_{My}$$ = 2.05965, $$C_{Mx}$$ = 1.54658, $$C_{Myx}$$ = 2.74295.

Population 2: [Source^29^].

*Y* = Oil prices in present week in 2017, *X* = Oil prices in preceding week from 2017.

*N* = 51, *n* = 11, $$\Lambda$$ = 0.01782, $${M}_{y}$$ = 25.80, $${M}_{x}$$ = 25.60, *fy* $$\left({M}_{y}\right)$$ = 0.0728, *fx* $$\left({M}_{x}\right)$$ = 0.1080, $${\rho }_{yx}$$ = 0.9956, $$C_{My}$$ = 0.53242, $$C_{Mx}$$ = 0.36168, $$C_{Myx}$$ = 0.19172.

Population 3: [Source^3^].

*Y* = Advanced degrees in 2007 of US, *X* = Advanced degrees in 2006 of US.

*N* = 1134, *n* = 210, $$\Lambda$$ = 0.00097, $${M}_{y}$$ = 48.55, $${M}_{x}$$ = 48.49, *fy* $$\left({M}_{y}\right)$$ = 0.0075349, *fx* $$\left({M}_{x}\right)$$ = 0.0075437, $${\rho }_{yx}$$ = 0.9953, $$C_{My}$$ = 2.73359, $$C_{Mx}$$ = 2.7378, $$C_{Myx}$$ = 7.43791.

## Empirical study

In this section, a simulation study was acknowledged to estimate the performance of the estimators empirically. Three populations are generated of size 1500 from normal population with theoretical means 5, for both *Y* and *X*. The variance covariance metrics used for three different populations are given by:

Population 1:

$$\sum =\left[\begin{array}{cc}12& 4\\ 4& 3\end{array}\right].$$

Population 2:

$$\sum =\left[\begin{array}{cc}8& 4\\ 4& 2\end{array}\right].$$

Population 3:

$$\sum =\left[\begin{array}{cc}10& 2.9\\ 2.9& 3\end{array}\right].$$

The sample sizes n = 50, 150, 250 are chosen in each population. The population density functions for *Y* and *X* are calculated utilizing normal distributions. Tables 3 and 4 comprise the mean square error and percentage relative efficiency based on the simulated data.

## Conclusion

In this article, we have suggested an enhanced estimator for estimation of population median under simple random sampling using auxiliary variable. Up to the first order of approximation, we evaluated into the bias and MSE of the suggested estimator. Three different datasets are used to evaluate the proposed generalized class of estimators against existing estimators. To check the novelty of estimator a simulation study is also conducted. The results of mean square error and percentage relative efficiency using actual data sets are given in Tables 1 and 2. Three simulated populations are generated of population size 1500 from normal distribution with theoretical means 5, for both *Y* and *X*. The sample sizes *n* = 50, 150, 250 are chosen in each population. The population density functions for *Y* and *X* are calculated utilizing normal distributions. The results based on simulation study include on Tables 3 and 4. Based on outcome of actual data sets and a simulation study, we can observe that the suggested estimator achieved minimum MSE and the higher percentage relative efficiency. This shows that the suggested estimator is more efficient for practical determinations when compared to other estimators. Consequently, regarding the new survey, we highly suggest choosing the suggested estimators discussed in this work for calculating the finite population median under the scenario of simple random sampling. The current work can be easily extended to estimation population mean using predictive approach under stratified and systematic random sampling. Further this work is easily extended to estimate population median under stratified random sampling.

**Table 1:** MSE result of all estimators considered in this article using real data sets.

Estimators	Population 1	Population 2	Population 3
$\hat{M}_{y}$	403,887.0	3.3633680	17.08538
$\hat{M}_{Rat}$	109,313.0	0.3467658	0.1606138
$\hat{M}_{\exp }$	199,668.0	1.4564990	4.3510590
$\hat{M}_{SS1}$	102,203.3	3.3165700	16.606740
$\hat{M}_{SS2}$	101,810.2	0.02953121	0.1602143
$\hat{M}_{D}$	104,407.5	0.02953252	0.1602252
$\hat{M}_{D1}$	103,797.0	0.02675913	0.1601913
$\hat{M}_{D2}$	103,116.3	0.02883137	0.1602002
$\hat{M}_{SS3}$	100,200.8	0.02945387	0.1579884
$\hat{M}_{Siraj(alpha = 0)}$	100,940.4	0.01838123	0.1139751
$\hat{M}_{Siraj(alpha = 1)}$	99,054.20	0.02929051	0.1415721
$\hat{M}_{proposed}$	90,648.43	0.02795507	0.1107349

**Table 2:** PRE result of all estimators considered in this article using real data sets.

Estimators	Population 1	Population 2	Population 3
$\hat{M}_{y}$	100	100	100
$\hat{M}_{Rat}$	369.4776	969.9249	10,637.55
$\hat{M}_{\exp }$	202.2793	230.9214	392.6718
$\hat{M}_{SS1}$	395.1800	101.41100	102.8822
$\hat{M}_{SS2}$	396.7058	11,389.200	10,664.08
$\hat{M}_{D}$	386.8372	11,388.69	10,663.35
$\hat{M}_{D1}$	389.1124	12,569.050	10,665.61
$\hat{M}_{D2}$	391.6810	11,665.65	10,665.02
$\hat{M}_{SS3}$	403.0776	11,419.10	10,814.33
$\hat{M}_{Siraj(alpha = 0)}$	400.1242	18,297.84	14,990.45
$\hat{M}_{Siraj(alpha = 1)}$	407.7434	11,482.79	12,068.32
$\hat{M}_{proposed}$	445.5532	12,031.33	15,429.08

**Table 3:** MSE result of all estimators considered in this article using simulated data.

Estimators		Population 1	Population 2	Population 3
		MSE	MSE	MSE
50	$\hat{M}_{y}$	0.3035376	0.3044098	0.3039826
	$\hat{M}_{Rat}$	0.2411791	0.1867652	0.3069938
	$\hat{M}_{\exp }$	0.2572681	0.2311382	0.2912059
	$\hat{M}_{SS1}$	0.2983535	0.2978961	0.2994347
	$\hat{M}_{SS2}$	0.2388759	0.1700244	0.2876469
	$\hat{M}_{D}$	0.2411627	0.1712726	0.2911662
	$\hat{M}_{D1}$	0.2389027	0.1707453	0.2869139
	$\hat{M}_{D2}$	0.2388705	0.1700213	0.2876386
	$\hat{M}_{SS3}$	0.2387305	0.1699169	0.2874722
	$\hat{M}_{Siraj(alpha = 0)}$	0.2395585	0.171118	0.2874365
	$\hat{M}_{Siraj(alpha = 1)}$	0.2384519	0.170344	0.2867422
	$\hat{M}_{proposed}$	0.2381115	0.1694469	0.2867414
100	$\hat{M}_{y}$	0.1468009	0.1465333	0.1465338
	$\hat{M}_{Rat}$	0.1142583	0.09100358	0.1530475
	$\hat{M}_{\exp }$	0.1234646	0.1114931	0.1426364
	$\hat{M}_{SS1}$	0.1454789	0.1451187	0.1455488
	$\hat{M}_{SS2}$	0.1135486	0.08471526	0.1414626
	$\hat{M}_{D}$	0.114096	0.08500341	0.1422657
	$\hat{M}_{D1}$	0.1135802	0.08486329	0.1412784
	$\hat{M}_{D2}$	0.113548	0.08471492	0.1414617
	$\hat{M}_{SS3}$	0.1135142	0.08469007	0.1414217
	$\hat{M}_{Siraj(alpha = 0)}$	0.1137391	0.08495595	0.1413882
	$\hat{M}_{Siraj(alpha = 1)}$	0.1134666	0.08476858	0.1412536
	$\hat{M}_{proposed}$	0.1133669	0.08457924	0.1412491
200	$\hat{M}_{y}$	0.06803388	0.06804364	0.06815512
	$\hat{M}_{Rat}$	0.05309361	0.04031044	0.07115151
	$\hat{M}_{\exp }$	0.05722006	0.05079539	0.06607069
	$\hat{M}_{SS1}$	0.06776494	0.0677253	0.06794988
	$\hat{M}_{SS2}$	0.05293588	0.03652598	0.06574962
	$\hat{M}_{D}$	0.0530478	0.03658026	0.06591406
	$\hat{M}_{D1}$	0.05294094	0.03656024	0.06570876
	$\hat{M}_{D2}$	0.05293582	0.03652595	0.06574953
	$\hat{M}_{SS3}$	0.05292872	0.03652084	0.06574056
	$\hat{M}_{Siraj(alpha = 0)}$	0.0529739	0.03657604	0.06573365
	$\hat{M}_{Siraj(alpha = 1)}$	0.05291756	0.0365417	0.06570288
	$\hat{M}_{proposed}$	0.05289791	0.03649803	0.06570201

**Table 4:** PRE result of all estimators considered in this article using simulated data.

Estimators		Population 1	Population 2	Population 3
		PRE	PRE	PRE
50	$\hat{M}_{y}$	100	100	100
	$\hat{M}_{Rat}$	125.8557	162.9906	99.01913
	$\hat{M}_{\exp }$	117.9849	131.7003	104.3875
	$\hat{M}_{SS1}$	101.7376	102.1866	101.5188
	$\hat{M}_{SS2}$	127.0692	179.0389	105.6791
	$\hat{M}_{D}$	125.8642	177.7341	104.4017
	$\hat{M}_{D1}$	127.0549	178.283	105.9491
	$\hat{M}_{D2}$	127.072	179.0422	105.6821
	$\hat{M}_{SS3}$	127.1466	179.1522	105.7433
	$\hat{M}_{Siraj(alpha = 0)}$	126.7071	177.8947	105.7564
	$\hat{M}_{Siraj(alpha = 1)}$	127.2951	178.703	106.0125
	$\hat{M}_{proposed}$	127.4771	179.6491	106.0128
100	$\hat{M}_{y}$	100	100	100
	$\hat{M}_{Rat}$	128.4816	161.0193	95.744
	$\hat{M}_{\exp }$	118.9012	131.4281	102.7324
	$\hat{M}_{SS1}$	100.9087	100.7248	100.7251
	$\hat{M}_{SS2}$	129.2846	172.9716	103.5848
	$\hat{M}_{D}$	128.6644	172.3852	103.0001
	$\hat{M}_{D1}$	129.2487	172.6698	103.7199
	$\hat{M}_{D2}$	129.2853	172.9722	103.5855
	$\hat{M}_{SS3}$	129.3238	173.023	103.6148
	$\hat{M}_{Siraj(alpha = 0)}$	129.0681	172.4815	103.6393
	$\hat{M}_{Siraj(alpha = 1)}$	129.3781	172.8628	103.7381
	$\hat{M}_{proposed}$	129.4919	173.2497	103.7414
200	$\hat{M}_{y}$	100	100	100
	$\hat{M}_{Rat}$	128.1395	168.7991	95.78872
	$\hat{M}_{\exp }$	118.8987	133.9563	103.1548
	$\hat{M}_{SS1}$	100.3969	100.47	100.302
	$\hat{M}_{SS2}$	128.5213	186.2883	103.6586
	$\hat{M}_{D}$	128.2501	186.0119	103.4
	$\hat{M}_{D1}$	128.509	186.1138	103.723
	$\hat{M}_{D2}$	128.5214	186.2885	103.6587
	$\hat{M}_{SS3}$	128.5387	186.3146	103.6729
	$\hat{M}_{Siraj(alpha = 0)}$	128.4291	186.0334	103.6838
	$\hat{M}_{Siraj(alpha = 1)}$	128.5658	186.2082	103.7323
	$\hat{M}_{proposed}$	128.6136	186.431	103.7337

## Data Availability

The datasets used and/or analysed during the current study available from the corresponding author on reasonable request.
